# Real-world effectiveness of fremanezumab in migraine patients initiating treatment in the United States: results from a retrospective chart study

**DOI:** 10.1186/s10194-022-01411-1

**Published:** 2022-04-11

**Authors:** Maurice T. Driessen, Joshua M. Cohen, Oscar Patterson-Lomba, Stephen F. Thompson, Michael Seminerio, Karen Carr, Todor I. Totev, Rochelle Sun, Erica Yim, Fan Mu, Rajeev Ayyagari

**Affiliations:** 1 Teva Pharmaceuticals, Piet Heinkade 107, 1019 BR Amsterdam, Netherlands; 2 Teva Branded Pharmaceutical Products R&D, Inc, West Chester, PA USA; 3 Analysis Group, Boston, MA USA; 4 Teva Branded Pharmaceuticals, Parsippany, NJ USA

**Keywords:** Fremanezumab, CGRP, Migraine preventive treatment, Real-world effectiveness, Chart review

## Abstract

**Background:**

The efficacy and tolerability of fremanezumab, a fully humanized monoclonal antibody (IgG2Δa) that selectively targets calcitonin gene-related peptide (CGRP) and is approved for the preventive treatment of migraine in adults, have been demonstrated in randomized, double-blind, placebo-controlled trials. Real-world data can further support those clinical trial data and demonstrate the full clinical benefits of fremanezumab. This chart review assessed the effectiveness of fremanezumab for improving clinical outcomes in adult patients with migraine treated according to real-world clinical practice.

**Methods:**

This retrospective, panel-based, online physician chart review study used electronic case report forms with US physicians. Patient inclusion criteria were a physician diagnosis of migraine, fremanezumab treatment initiation at ≥ 18 years of age after US Food and Drug Administration approval, ≥ 1 dose of fremanezumab treatment, and ≥ 2 assessments of monthly migraine days (MMD; 1 within 30 days before treatment initiation and ≥ 1 after initiation). Changes from baseline in MMD, monthly headache days (MHD), and Migraine Disability Assessment (MIDAS) and 6-item Headache Impact Test (HIT-6) scores were assessed over 6 months. These endpoints were evaluated in the overall population and subgroups divided by dosing schedule and number of prior migraine preventive treatment failures.

**Results:**

This study included data from 421 clinicians and 1003 patients. Mean age at fremanezumab initiation was 39.7 years, and most patients were female (75.8%). In the overall population, mean baseline MMD and MHD were 12.7 and 14.0, respectively. Mean (percent) reductions from baseline in MMD and MHD, respectively, were − 4.6 (36.2%) and − 4.7 (33.6%) at Month 1, − 6.7 (52.8%) and − 6.8 (48.6%) at Month 3, and − 9.2 (72.4%) and − 9.8 (70.0%) at Month 6. Mean (percent) reductions from baseline in MIDAS and HIT-6 scores also increased over the 6-month study period, from − 6.2 (21.6%) and − 8.4 (14.0%) at Month 1 to − 18.1 (63.1%) and − 16.2 (27.0%) at Month 6, respectively. Improvements in these outcomes over 6 months were observed across all evaluated subgroups.

**Conclusions:**

This real-world study demonstrated effectiveness of fremanezumab treatment for up to 6 months, irrespective of dosing regimen or number of prior migraine preventive treatment failures, reflecting ongoing, clinically meaningful improvements in patient outcomes.

**Supplementary Information:**

The online version contains supplementary material available at 10.1186/s10194-022-01411-1.

## Background

Migraine, 1 of the 3 most burdensome neurological diseases in the United States, affects approximately 68.5 million people and results in > 2 million disability-adjusted life-years annually [1]. Migraine is associated with a substantial negative impact on health-related quality of life and daily function. In an analysis of data from the US 2016 National Health and Wellness Survey (NHWS) in patients with migraine reporting ≥ 4 headache days per month, patients experienced substantial activity impairment, lost work productivity, and increased healthcare resource utilization compared with non-migraine controls [2]. Migraine also has a negative effect on patients’ family lives. In the Chronic Migraine Epidemiology and Outcomes (CaMEO) study, US patients with migraine reported reduced participation in and enjoyment of family activities, impaired parenting ability, and concerns about their family’s financial security due to their headaches [3]. The analysis of the NHWS data also demonstrated improvements in patients’ participation in home activities and reductions in work absenteeism and associated indirect costs with each incremental increase in headache-free days [4].

Appropriate treatment of migraine may increase the number of headache-free days for patients, thereby reducing the burden and consequences of migraine [4]. Unfortunately, migraine is often undertreated or underdiagnosed [5]. Some migraine preventive treatment options include antihypertensives, antiepileptics, antidepressants, calcium channel blockers, and onabotulinumtoxinA [6]. However, these treatments are not primarily indicated for migraine and may have limited efficacy or poor tolerability, leading patients to switch or discontinue treatment altogether [7–9].

Fremanezumab, a fully humanized monoclonal antibody (IgG2Δa) that selectively targets calcitonin gene-related peptide (CGRP), has been approved by the US Food and Drug Administration (FDA) for the preventive treatment of migraine in adults [10]. The safety and efficacy of 2 dosing regimens of fremanezumab (quarterly and monthly) in adults with chronic migraine (CM; ≥ 15 headache days per month) and episodic migraine (EM; 6–14 headache days per month), including in patients with inadequate response to 2 to 4 prior migraine preventive treatment classes, have been demonstrated in randomized, double-blind, placebo-controlled, phase 3 studies (HALO CM, HALO EM, and FOCUS) [11–13]. The long-term efficacy and safety of fremanezumab have been demonstrated in a randomized, double-blind, 12-month extension study [14]. Real-world data on the clinical use of fremanezumab and other CGRP pathway–targeted monoclonal antibodies is limited [15–19]. Thus, there is a need for real-world effectiveness data to support the findings of clinical studies of fremanezumab [20]. The current retrospective, panel-based, online physician chart review aimed to assess the effectiveness of fremanezumab in adult patients with migraine up to 6 months.

## Methods

### Study design

**Fig. 1:**
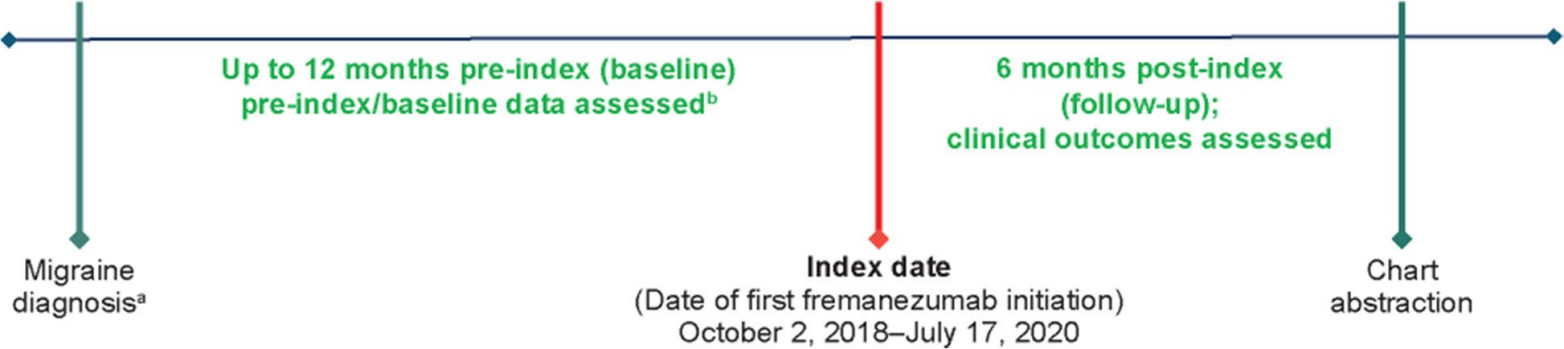
Study design and dosing for patients with migraine. EM, episodic migraine; CM, chronic migraine; MMD, monthly migraine days; MHD, monthly headache days; HIT-6, 6-item Headache Impact Test; MIDAS, Migraine Disability Assessment. ^a^Migraine diagnosis could have been established before 12 months pre-index, but data were extracted as close to fremanezumab initiation as possible. ^b^Baseline patient information (eg, comorbidities, prior treatments) were collected over 12 months pre-index, while baseline clinical outcomes (eg, MMD, MHD, HIT-6, MIDAS) were collected during the 3 months pre-index

This was a retrospective, online, panel-based clinician survey conducted in the United States with an integrated electronic case report form (eCRF) for the chart review. The clinician survey was used to confirm clinician eligibility and collect information on clinicians’ demographic and practice characteristics. Data collection was double-blind so that physicians were not aware of the funding party, and the sponsor was not made aware of which physicians participated. An initial data assessment among 50 physicians treating patients with migraine evaluated the availability and quality of certain data elements of interest, and this assessment informed the study design. The physician panel-based chart review occurred in 3 stages, which included a pilot test and a soft launch to ensure clarity of the eCRF questions and for quality assurance purposes, followed by a full launch. Clinicians were then provided the custom-designed eCRF to enter data from eligible patient charts, including demographic characteristics and migraine assessments (within 1 month of first fremanezumab initiation), clinical characteristics and prior treatment patterns (within 12 months of first fremanezumab initiation), and incidence of psychiatric comorbidities (within 3 months of first fremanezumab initiation). Study outcomes were evaluated during the full 6-month follow-up period after fremanezumab initiation until treatment discontinuation or chart abstraction (Fig. 1). The date of first fremanezumab initiation was designated as the index date.

### Physician and patient eligibility criteria

Eligible clinicians included neurologists, general practitioners, pain management specialists, psychiatrists, physician assistants, nurse practitioners, and other headache specialists routinely treating patients with migraine in a US-based practice. Clinicians were also required to have personally treated ≥ 5 adult patients diagnosed with migraine, including patients meeting the inclusion criteria, in the past 12 months.

Clinicians were sampled using a tiered approach in an effort to maximize the number of neurologists and pain management specialist respondents, as follows: tier 1, neurologists; tier 2, pain management specialists; and tier 3, general practitioners (family practice, general practice, and primary care physicians), nurse practitioners, physician assistants, and psychiatrists.

Participating clinicians selected and completed ≤ 5 charts for patients who met all of the following inclusion criteria: physician diagnosis of migraine; first fremanezumab treatment initiation at ≥ 18 years of age, after FDA approval (September 14, 2018) and their diagnosis of migraine; not pregnant in the 12 months prior to or during fremanezumab treatment; treated with ≥ 1 dose of fremanezumab; ≥ 1 follow-up since fremanezumab treatment initiation; and chart containing ≥ 2 monthly migraine day (MMD) measurements (1 at fremanezumab initiation and ≥ 1 during fremanezumab treatment). To minimize selection bias, physicians were instructed to identify all charts meeting the selection criteria and to randomly select up to 5 of those charts for data extraction using a randomization scheme. For randomization, the eCRF had an underlying program that required clinicians select patient charts based on a randomized sequence of letters. For example, the program produced a random letter and requested clinicians pull a patient chart with a last name corresponding to the random letter. The random process repeated for each new patient chart completed by the clinician.

### Outcome assessment

Outcomes were evaluated in patients with migraine in the overall population, as well as in subgroups divided by dosing regimen (quarterly or monthly fremanezumab dosing) and by number of prior preventive treatment failures (< 2, ≥ 2, 2 to 4, and > 4 prior failures). Treatment discontinuations were assessed in the overall population. The changes from baseline in MMD and monthly headache days (MHD) were evaluated at each monthly timepoint, along with the proportion of patients with a clinically meaningful ≥ 30%, ≥ 50%, or ≥ 75% reduction in MMD. Headache- and migraine-related disability were evaluated using validated measures: the 6-item Headache Impact Test (HIT-6) [21] and Migraine Disability Assessment (MIDAS) [22], respectively. Total HIT-6 scores range from 36 to 78, with 4 impact severity categories: little or no impact (≤ 49), some impact (50–55), substantial impact (56–59), and severe impact (60–78) [21]. Total MIDAS scores indicate the severity of disability: little or no disability (0–5), mild disability (6–10), moderate disability (11–20), and severe disability (≥ 21) [22]. Clinically meaningful changes are defined as a ≥ 5-point and a ≥ 6-point decrease for HIT-6 and MIDAS scores, respectively [23, 24]. Adherence and persistence (time to treatment discontinuation) were reported over the full follow-up period, the length of which varied among patients. To address the heterogeneity in the assessment times observed in real-world practice, the average monthly outcomes (ie, MMD, MHD, and HIT-6 and MIDAS scores) were calculated among patients with data reported within ± 15 days of each timepoint (eg, Month 1, Month, 2). Due to this heterogeneity in the assessment times, not all patients were included in the analyses for each timepoint and sample sizes varied accordingly.

### Statistical analysis

The sample size of the subgroups was not based on any statistical considerations. A sample of 1000 patient charts was targeted to ensure sufficient sample sizes among patient subgroups to detect reductions in the mean number of MMD and MHD comparable to those observed in the FOCUS study, assuming 80% power and 5% type 1 error rate [12]. Outcome variables were summarized using descriptive statistics (mean and standard deviation [SD] or median and interquartile range) for continuous variables and frequency distributions for categorical variables. Patient characteristics and effectiveness and disability outcomes were evaluated in the patient subgroups, and results were compared between pairs of relevant stratifications using chi-squared tests for categorical variables and either Wilcoxon rank sum or *t-*tests for continuous variables. All analyses were conducted in SAS Enterprise Guide, version 7.1 or higher (SAS Institute, Cary, NC), and R, version 3.5.1 or higher (R Foundation for Statistical Computing, Vienna, Austria).

## Results

### Patient characteristics

This study included data from 421 clinicians and 1003 patients. The majority of clinicians were neurologists (57.0% [240/421]) and general practitioners (19.0% [80/421]), and clinicians had been in practice for a mean (SD) of 14.7 (8.7) years. Clinicians had treated a mean (SD) of 367.0 (457.6) patients with migraine within the last 12 months and were treating a mean (SD) of 68.1 (159.2) patients with migraine with fremanezumab (Additional file 1). Of the 1003 patients included, 760 (75.8%) were female, the mean (SD) age was 39.7 (12.4) years, and the mean (SD) time from diagnosis to index date was 6.6 (8.5) years (Table 1). At baseline, common comorbid conditions included insomnia (196 [19.5%]), hypertension (143 [14.3%]), major depressive disorder (134 [13.4%]), and generalized anxiety disorder (120 [12.0%]). For the dosing subgroups, 381 (38.0%) and 622 (62.0%) patients were treated with quarterly and monthly fremanezumab, respectively. A total of 171 (17.0%), 832 (83.0%), 689 (68.7%), and 143 (14.3%) patients had < 2, ≥ 2, 2 to 4, and > 4 prior preventive treatment failures, respectively.

**Table 1:** Patient Baseline and Demographic Characteristics

Baseline and demographic characteristics	All patients (*n* = 1003)
Age at index date, years, mean (SD)	39.7 (12.4)
Sex, n (%)	
Female	760 (75.8)
Male	241 (24.0)
Other	2 (0.2)
Race, n (%)	
White	782 (78.0)
Black or African American	137 (13.7)
Asian	56 (5.6)
Native American or American Indian	7 (0.7)
Other	20 (2.0)
Unknown	1 (0.1)
Migraine diagnosis, n (%)	
CM	587 (58.5)
EM	416 (41.5)
Duration of follow-up (months), mean (SD)	7.1 (4.4)
Time from date of diagnosis to index date (years), mean (SD)	6.6 (8.5)
Number of prior treatment failures, n (%)	
0	60 (6.0)
1	111 (11.1)
≥ 2	832 (82.9)
Baseline MMD, mean (SD)	12.7 (6.4)
Baseline MHD, mean (SD)	14.0 (8.0)
Baseline HIT-6 score, mean (SD)	60.0 (8.9)
Baseline MIDAS score, mean (SD)	28.7 (28.5)
Comorbid conditions at baseline, n (%)^a^	
Insomnia	196 (19.5)
Allergies	158 (15.8)
Neck pain	155 (15.5)
Hypertension	143 (14.3)
Back pain	138 (13.8)
Obesity	137 (13.7)
MDD	134 (13.4)
GAD	120 (12.0)
Chronic pain	111 (11.1)
Asthma	101 (10.1)
Fibromyalgia	96 (9.6)
High cholesterol	94 (9.4)
Gastroesophageal reflux	80 (8.0)
Other anxiety (non-GAD)	60 (6.0)
Sinusitis	52 (5.2)

*CM* Chronic migraine, *EM* Episodic migraine, *SD* Standard deviation, *MMD* Monthly migraine days, *MHD* Monthly headache days, *HIT-6* 6-item Headache Impact Test, *MIDAS* Migraine Disability Assessment, *MDD* Major depressive disorder, *GAD* Generalized anxiety disorder

^a^Reported by ≥ 5% of patients

Overall, only 7.8% (78/1003) of patients discontinued fremanezumab treatment during the 6 months post index. Discontinuation rates during this timeframe in the monthly and quarterly dosing subgroups were 9.6% (60/622) and 4.7% (18/381), respectively. By prior treatment failures, discontinuation rates were 10.5% (18/171), 7.2% (60/832), 6.7% (46/689), and 9.8% (14/143) in the < 2, ≥ 2, 2 to 4, and > 4 prior treatment failures subgroups, respectively.

In the 12 months before first fremanezumab initiation, the most commonly used acute medications were anti-migraine analgesics (triptans or ergots; 72.2% [724/1003]), nonsteroidal anti-inflammatory drugs (NSAIDs; 60.4% [606/1003]), butalbital-containing compounds (32.1% [322/1003]), and narcotics/opioid analgesics (21.1% [212/1003]). The most commonly used preventive medications prior to starting fremanezumab treatment were antiepileptics or anticonvulsants (65.2% [654/1003]); antidepressants (54.5% [547/1003]); antihypertensives, antianginals, antiarrhythmics, and α-antagonists (41.5% [416/1003]); muscle relaxants (27.9% [280/1003]); and onabotulinumtoxinA (20.2% [203/1003]). A total of 9.8% (98/1003) of patients had prior exposure to another CGRP pathway–targeted monoclonal antibody treatment. During the study period, the most commonly used acute medications were anti-migraine analgesics (48.9% [490/1003]) and NSAIDs (38.6% [387/1003]), while the most commonly used preventive medications were antidepressants (18.4% [185/1003]) and antiepileptics or anticonvulsants (18.0% [181/1003]).

### Reductions in MMD

#### Overall population

**Fig. 2:**
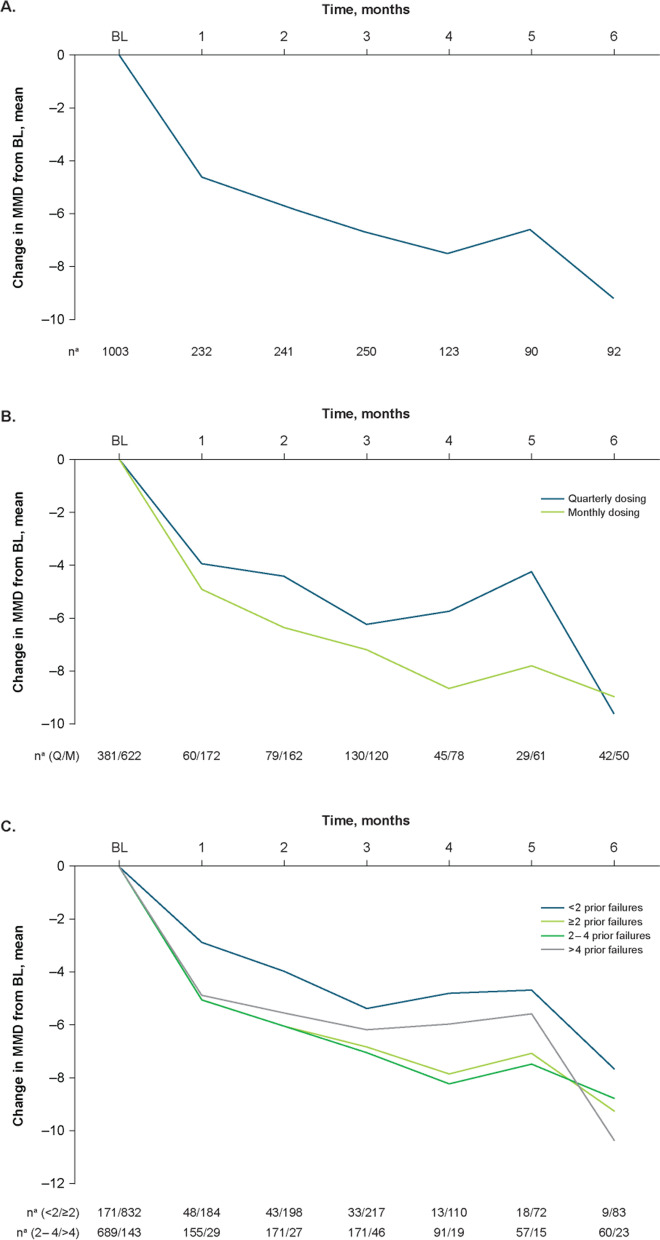
Effectiveness outcomes by change from baseline in MMD: **A**) total population; **B**) dosing schedule subgroups; **C**) prior treatment failures subgroups. BL, baseline; MMD, monthly migraine days; Q, quarterly; M, monthly. ^a^Number of patients with available assessment at each time point

**Fig. 3:**
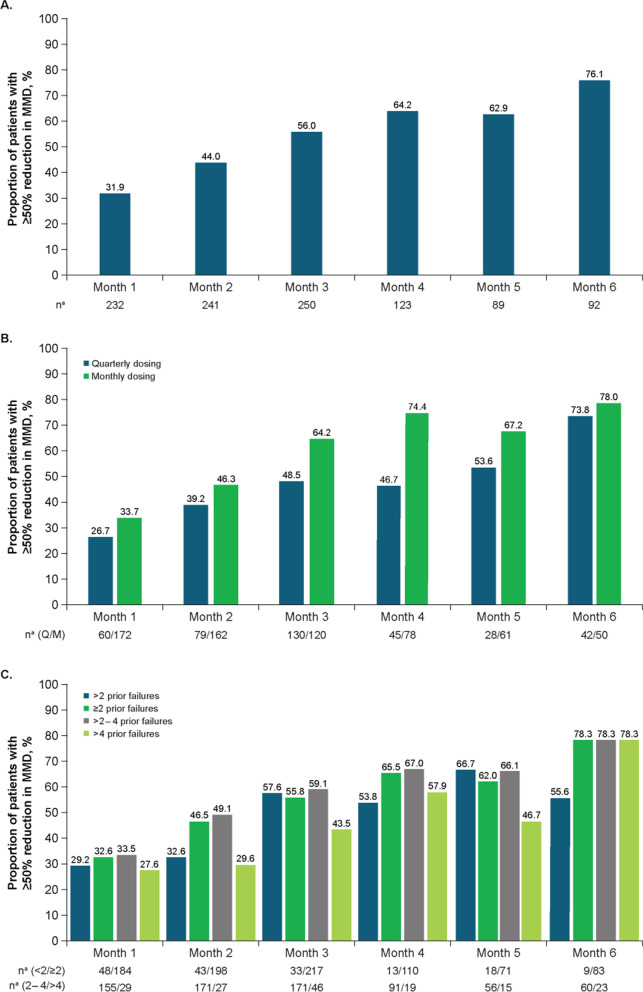
Effectiveness outcomes by proportion of patients with a ≥ 50% reduction from baseline in MMD: **A**) total population; **B**) dosing schedule subgroups; **C**) prior treatment failures subgroups. BL, baseline; MMD, monthly migraine days; Q, quarterly; M, monthly. ^a^Number of patients with available assessment at each time point

In the overall population, the baseline mean (SD) number of MMD was 12.7 (6.4), and mean (percent) reductions from baseline were − 4.6 (36.2%) at Month 1, − 6.7 (52.8%) at Month 3, and − 9.2 (72.4%) at Month 6 (Fig. 2A). The proportion of patients with a ≥ 50% reduction in MMD increased from Month 1 (31.9% [74/232]) to Month 6 (76.1% [70/92]; Fig. 3A), as did the proportion of patients with a ≥ 30% reduction in MMD (Month 1, 56.0% [130/232]; Month 6, 89.1% [82/92]; Additional file 2A) and those with a ≥ 75% reduction in MMD (Month 1, 12.5% [29/232]; Month 6, 37.0% [34/92]; Additional file 3A).

#### Quarterly and monthly dosing

In the subgroups divided by dosing schedule at initiation, the mean (SD) baseline number of MMD was 11.9 (6.1) in the quarterly fremanezumab subgroup and 13.2 (6.5) in the monthly fremanezumab subgroup. Mean (percent) reductions in MMD from baseline increased over 6 months with both quarterly and monthly dosing, respectively, from Month 1 (− 3.9 [32.8%] and − 4.9 [37.1%]) to Month 6 (− 9.5 [79.8%] and − 8.9 [67.4%]; Fig. 2B). The proportion of patients with a ≥ 50% reduction in MMD increased from Month 1 (26.7% [16/60]) to Month 6 (73.8% [31/42]) in the quarterly fremanezumab subgroup and from Month 1 (33.7% [58/172]) to Month 6 (78.0% [39/50]) in the monthly fremanezumab subgroup (Fig. 3B). Similarly, the proportion of patients with a ≥ 30% reduction in MMD increased in both the quarterly and monthly dosing subgroups, respectively, from Month 1 (53.3% [32/60] and 57% [98/172]) to Month 6 (85.7% [36/42] and 92% [46/50]; Additional file 2B), as did those with a ≥ 75% reduction in MMD (Month 1, 6.7% [4/60] and 14.5% [25/172]; Month 6, 31.0% [13/42] and 42.0% [21/50]; Additional file 3B).

####  < *2,* ≥ *2, 2 to 4, and* > *4 Prior migraine preventive treatment failures*

In the subgroups divided by < 2 and ≥ 2 prior migraine preventive treatment failures, mean (SD) baseline numbers of MMD in the < 2 prior failures and ≥ 2 prior failures subgroups, respectively, were 10.5 (6.3) and 13.2 (6.3). Mean (percent) reductions from baseline in MMD for the < 2 prior failures subgroup increased from − 2.9 (27.6%) at Month 1 to − 7.8 (74.3%) at Month 6, and mean (percent) reductions from baseline in MMD for the ≥ 2 prior failures subgroup were − 5.1 (38.6%) at Month 1 and − 9.4 (71.2%) at Month 6 (Fig. 2C). Additionally, mean (SD) baseline MMD in the 2 to 4 and > 4 prior failures subgroups, respectively, were 13.1 (6.3) and 13.6 (6.4). Mean (percent) reductions from baseline in MMD for the 2 to 4 prior failures subgroup increased from − 5.1 (38.9%) at Month 1 to − 8.9 (67.9%) at Month 6, and mean (percent) reductions from baseline in MMD for the > 4 prior failures subgroup increased from − 4.9 (36.0%) at Month 1 and − 10.7 (78.7%) at Month 6. The proportion of patients with a ≥ 50% reduction in MMD increased over 6 months in the < 2 prior failures subgroup (Month 1, 29.2% [14/48]; Month 6, 55.6% [5/9]) and ≥ 2 prior failures subgroup (Month 1, 32.6% [60/184]; Month 6, 78.3% [65/83]; Fig. 3C), as did the proportion with a ≥ 30% reduction in MMD in both the < 2 and ≥ 2 prior failures subgroups (Month 1, 54.2% [26/48] and 56.5% [104/184], respectively; Month 6, 88.9% [8/9] and 89.2% [74/83], respectively; Additional file 2C) and those with a ≥ 75% reduction in MMD (Month 1, 4.2% [2/48] and 14.7% [27/184], respectively; Month 6, 33.3% [3/9] and 37.3% [31/83], respectively; Additional file 3C). Likewise, the proportion of patients with a ≥ 50% reduction in MMD increased over 6 months in the 2 to 4 prior failures subgroup (Month 1, 33.5% [52/155]; Month 6, 78.3% [47/60]) and the > 4 prior failures subgroup (Month 1, 27.6% [8/29]; Month 6, 78.3% [18/23]; Fig. 3C), as did the proportion with a ≥ 30% reduction in MMD in both the 2 to 4 and > 4 prior failures subgroups, respectively (Month 1, 56.1% [87/155] and 58.6% [17/29]; Month 6, 86.7% [52/60] and 95.7% [22/23]; Additional file 2C) and those with a ≥ 75% reduction in MMD (Month 1, 14.2% [22/155] and 17.2% [5/29], respectively; Month 6, 38.3% [23/60] and 34.8% [8/23], respectively; Additional file 3C).

### Reductions in MHD

#### Overall population

**Fig. 4:**
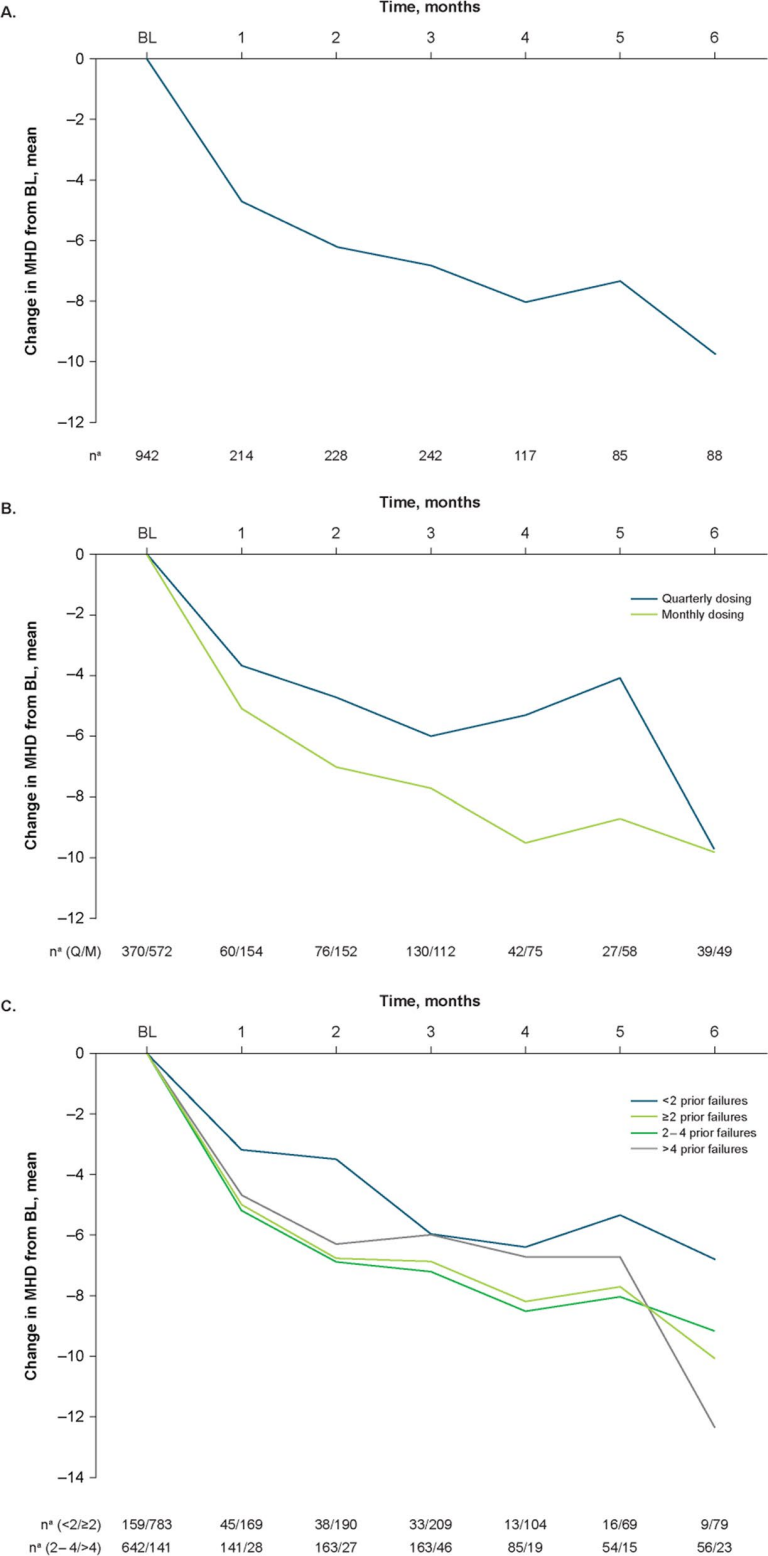
Effectiveness outcomes by change from baseline in MHD: **A**) total population; **B**) dosing schedule subgroups; **C**) prior treatment failures subgroups. BL, baseline; MHD, monthly headache days; Q, quarterly; M, monthly. ^a^Number of patients with available assessment at each time point

In the overall population, the baseline mean (SD) number of MHD was 14.0 (8.0), and mean (percent) reduction from baseline was − 4.7 (33.6%) at Month 1, − 6.8 (48.6%) at Month 3, and − 9.8 (70.0%) at Month 6 (Fig. 4A).

#### Quarterly and monthly dosing

In the subgroups divided by dosing schedule at initiation, the baseline mean (SD) number of MHD was 12.9 (7.7) in the quarterly fremanezumab subgroup and 14.8 (8.0) in the monthly fremanezumab subgroup. Mean (percent) reductions in MHD from baseline increased over 6 months with both quarterly and monthly dosing, respectively, from Month 1 (− 3.7 [28.7%] and − 5.1 [34.5%]) to Month 6 (− 9.7 [75.2%] and − 9.8 [66.2%]; Fig. 4B).

####  < 2, ≥ 2, 2 to 4, and > 4 Prior migraine preventive treatment failures

Mean (SD) baseline MHD for the < 2, ≥ 2, 2 to 4, and > 4 prior preventive treatment failures subgroups, respectively, were 11.0 (7.2), 14.6 (8.0), 14.2 (7.8), and 16.4 (8.6). Mean (percent) reductions from baseline in MHD increased from − 3.2 (29.1%) at Month 1 to − 6.8 (61.8%) at Month 6 for the < 2 prior failures subgroup and from − 5.1 (34.9%) at Month 1 to − 10.1 (69.2%) at Month 6 to the ≥ 2 prior failures subgroup (Fig. 4C). Moreover, mean (percent) reductions from baseline in MHD for the 2 to 4 prior failures subgroup increased from − 5.2 (36.6%) at Month 1 to − 9.2 (64.8%) at Month 6, and for the > 4 prior failures subgroup, from − 4.7 (28.7%) at Month 1 to − 12.4 (75.6%) at Month 6 (Fig. 4C).

### Reductions in HIT-6 scores

#### Overall population

**Fig. 5:**
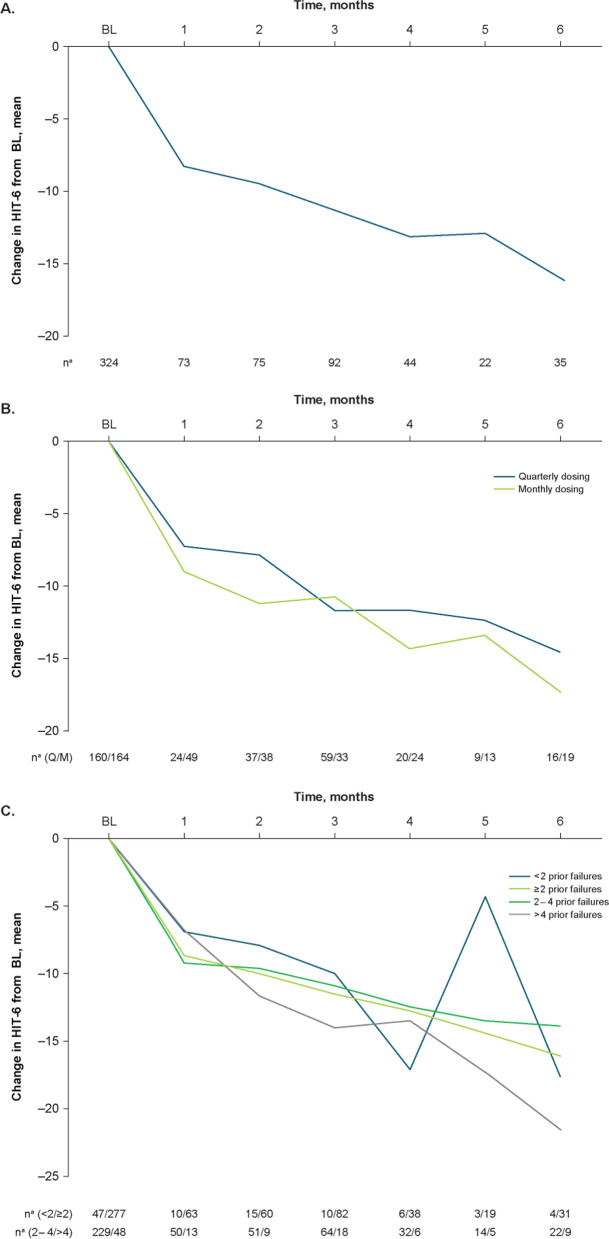
Effectiveness outcomes by change from baseline in HIT-6 scores: **A**) total population; **B**) dosing schedule subgroups; **C**) prior treatment failures subgroups. BL, baseline; HIT-6, 6-item Headache Impact Test; Q, quarterly; M, monthly. ^a^Number of patients with available assessment at each time point

In the overall population, the baseline mean (SD) HIT-6 score was 60.0 (8.9). Mean (percent) reductions from baseline in HIT-6 scores were − 8.4 (14.0%) at Month 1 and − 16.2 (27.0%) at Month 6 (Fig. 5A).

#### Quarterly and monthly dosing

Mean (SD) baseline HIT-6 scores for the quarterly and monthly fremanezumab subgroups were 60.1 (9.0) and 59.9 (8.7), respectively. Mean (percent) reductions from baseline in HIT-6 scores in the quarterly and monthly dosing subgroups, respectively, were − 7.3 (12.1%) and − 9.0 (15.0%) at Month 1 and − 14.7 (24.5%) and − 17.4 (29.0%) at Month 6 (Fig. 5B).

####  < 2, ≥ 2, 2 to 4, and > 4 Prior migraine preventive treatment failures

Mean (SD) baseline HIT-6 scores for the < 2 and ≥ 2 prior preventive treatment failures subgroups, respectively, were 56.3 (7.7) and 60.6 (8.9), and for the 2 to 4 and > 4 prior preventive treatment failures subgroups, respectively, were 59.9 (8.8) and 63.9 (8.5). Mean (percent) reductions from baseline in the < 2 and ≥ 2 prior failures subgroups, respectively, were − 6.8 (12.1%) and − 8.7 (14.4%) at Month 1 and − 17.5 (31.1%) and − 16.0 (26.4%) at Month 6. Mean (percent) reductions from baseline in the 2 to 4 and > 4 prior failures subgroups, respectively, were − 9.2 (15.4%) and − 6.9 (10.8%) at Month 1 and − 13.8 (23.0%) and − 21.4 (33.5%) at Month 6 (Fig. 5C).

### Reductions in MIDAS Scores

#### Overall population

**Fig. 6:**
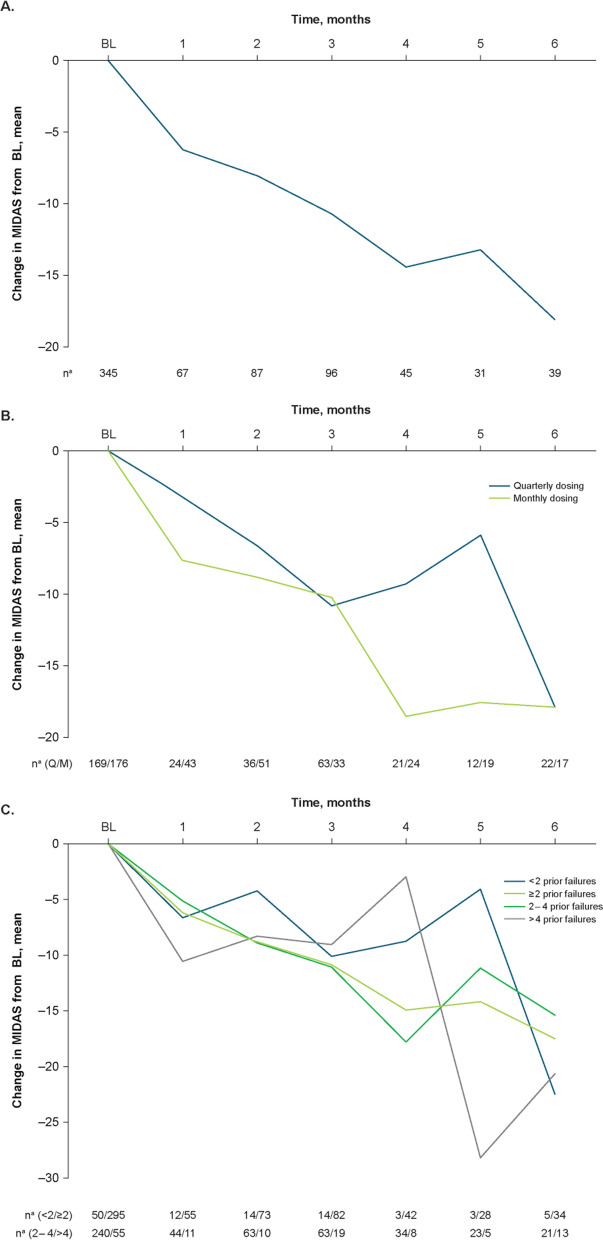
Effectiveness outcomes by change from baseline in MIDAS score: **A**) total population; **B**) dosing schedule subgroups; **C**) prior treatment failures subgroups. BL, baseline; MIDAS, Migraine Disability Assessment; Q, quarterly; M, monthly. ^a^Number of patients with available assessment at each time point

In the overall population, the baseline mean (SD) MIDAS score was 28.7 (28.5). Mean (percent) reductions from baseline in MIDAS scores were − 6.2 (21.6%) at Month 1 and − 18.1 (63.1%) at Month 6 (Fig. 6A).

#### Quarterly and monthly dosing

Mean (SD) baseline MIDAS scores in the quarterly and monthly fremanezumab subgroups were 29.6 (23.2) and 27.9 (32.9), respectively. Mean (percent) reductions from baseline in the quarterly and monthly dosing subgroups, respectively, increased from − 3.2 (10.8%) and − 7.9 (28.3%) at Month 1 to − 18.1 (61.1%) and − 18.0 (64.5%) at Month 6 (Fig. 6B).

####  < 2, ≥ 2, 2 to 4, and > 4 Prior migraine preventive treatment failures

The mean (SD) baseline MIDAS scores were 28.7 (25.1) and 28.7 (29.1) in the < 2 and ≥ 2 prior preventive treatment failures subgroups, respectively. Mean (percent) reductions from baseline in MIDAS scores in the < 2 and ≥ 2 prior preventive treatment failures subgroups, respectively, were − 6.6 (23.0%) and − 6.1 (21.3%) at Month 1 and − 22.4 (78.0%) and − 17.4 (60.6%) at Month 6 (Fig. 6C). In the 2 to 4 and > 4 prior preventive treatment failures subgroups, mean (SD) baseline MIDAS scores were 28.9 (28.7) and 28.1 (31.3), respectively, and mean (percent) reductions from baseline in MIDAS scores were − 5.1 (17.6%) and − 10.5 (37.4%) at Month 1 and − 15.4 (53.3%) and − 20.7 (73.7%) at Month 6 (Fig. 6C).

## Discussion

This retrospective online panel-based physician chart review demonstrated the real-world effectiveness of fremanezumab based on reductions in MMD and MHD and improvements in disability outcomes over 6 months in a US real-world adult population with physician-diagnosed migraine who were initiating fremanezumab treatment. Effectiveness, based on those same outcomes, was also demonstrated across different subgroups of patients with migraine, including subgroups divided by the fremanezumab dosing schedule they were prescribed at initiation (quarterly or monthly) and their number of prior preventive treatment failures (< 2, ≥ 2, 2–4, and > 4).

Real-world studies like this one are typically more inclusive than clinical studies and involve a broader patient population. Whereas clinical trials are crucial to understand the efficacy and safety of treatment in specific patient populations in a controlled setting, the population in clinical trials of migraine preventive treatments are generally of a limited age range, may have fewer comorbidities, and may not adequately represent the general population of patients with migraine. Conducted outside of the typical controlled conditions of clinical trials, real-world effectiveness trials have been recognized by regulatory bodies as extremely useful complements to their parallel randomized trials [25]. These real-world studies offer insight across a more encompassing, diverse group of patients, which helps healthcare providers to make more well-informed decisions for the patients they see in clinical practice.

Across 2 pivotal randomized, double-blind, placebo-controlled, phase 3 HALO studies in patients with CM or EM, fremanezumab treatment resulted in significant reductions compared with placebo in MMD and MHD, and disability outcomes over 3 months of treatment (*P* ≤ 0.002); however, patients were not eligible for inclusion in those studies if they had failed ≥ 2 prior migraine preventive treatments [11, 13]. The randomized, double-blind, placebo-controlled, phase 3b FOCUS study included a more similar population to the current real-world study: patients with CM and EM with inadequate response to 2 to 4 prior classes of migraine preventive treatments [12]. In that study, fremanezumab treatment resulted in significant reductions compared with placebo in MMD, MHD, and disability outcomes (HIT-6 and MIDAS scores) over 3 months of treatment (*P* ≤ 0.0001) [12]. In the current study, the majority of patients (≥ 80%) had ≥ 2 prior preventive treatment failures, but the study includes data on patients with < 2, 2 to 4, and > 4 prior treatment failures as well. Average MMD at baseline were higher in the FOCUS study (14.1 in both the quarterly and monthly fremanezumab groups) than in the current real-world study (quarterly fremanezumab, 11.9; monthly fremanezumab, 13.2). However, average reductions in MMD were greater at 3 months in the current study (quarterly fremanezumab, − 6.2 [52% reduction]; monthly fremanezumab, − 7.2 [55%]) than over 3 months in the FOCUS study (quarterly fremanezumab, − 3.7 [26%]; monthly fremanezumab, − 4.1 [27%]) [12]. Mean baseline HIT-6 scores were comparable in the FOCUS study (quarterly fremanezumab, 64.2; monthly fremanezumab, 63.9) and the current real-world study (60.1 and 59.9, respectively), while baseline MIDAS scores were substantially lower (real-world study, 29.6 and 27.9, respectively; FOCUS study, 62.3 and 61.5, respectively) [12]. In both the quarterly and monthly fremanezumab dosing groups, respectively, reductions in HIT-6 scores with treatment were greater in the current study at 3 months (− 11.7 [19% reduction] and − 10.8 [18%]) than in the FOCUS study (− 6.1 [10%] and − 5.2 [8%]). Reductions in average MIDAS scores were lower in the current study at 3 months (quarterly fremanezumab, − 10.9; monthly fremanezumab, − 10.3) than in the FOCUS study (− 19.7 and − 24.7, respectively) [12]; however, percent reductions were generally comparable in the current study (37% in both dosing groups) and the FOCUS study (32% and 40%, respectively). Clinically meaningful reductions in both disability outcomes, HIT-6 and MIDAS scores, were observed as early as 1 month after treatment initiation in the overall population in the current study [23, 24].

Fremanezumab was efficacious in both the current study and the FOCUS study, regardless of number of prior treatment failures. The FOCUS study demonstrated similar efficacy and improvements in disability outcomes in patients with 2, 3, and 4 prior treatment failures [26] as in the current study in patients with 2 to 4 prior treatment failures. Moreover, in the current study, meaningful reductions in MMD were observed for patients with > 4 prior treatment failures, who were not included in either the FOCUS or the HALO studies, and both quarterly and monthly dosing regimens were found to be effective in improving clinical outcomes. Altogether, larger improvements were observed in the current study versus the outcomes from these various clinical studies of patients with migraine. These data show that fremanezumab is effective for an even broader population of patients with difficult-to-treat migraine.

Different outcomes between real-world and clinical trials have also been reported for other CGRP pathway–targeted monoclonal antibodies, erenumab and galcanezumab. In a real-world study of erenumab that included largely patients with CM (94%), all of whom had ≥ 2 prior preventive treatment failures, median MMD decreased by − 12 at 3 months with erenumab treatment [19]. In a separate subgroup analysis of patients with ≥ 2 prior failures from a randomized, double-blind, placebo-controlled, phase 2 trial, average reductions in MMD were lower, ranging from − 5.4 to − 7.0 by Month 3 [27]. Another study investigating the effects of erenumab in primarily patients with CM with complex comorbidities and refractory migraine found the average MMD reduction at 6 months to be − 8.6 [16]. In an open-label study, patients with CM taking erenumab achieved MMD reductions ranging from − 7.6 to − 11.5 after 52 weeks of therapy [28]. In another prospective, observational study of galcanezumab or erenumab treatment in patients with migraine with ≥ 8 headache days per month and ≥ 3 prior preventive medication failures, after 3 months of treatment, there was a ≥ 50% reduction in MMD in 51.6% of patients (mean [SD] reduction in MMD compared with baseline was − 8.5 [7.7]) [29]. A separate study involving patients with high-frequency CM and EM found that treatment with galcanezumab for 3 months resulted in a significant MMD reduction compared with baseline (EM, − 8.5; CM, − 11.5; both *P* < 0.0001) [30]. Furthermore, data on patients with CM who were prescribed erenumab were collected from US headache centers for a multicenter chart review. Results from that retrospective study demonstrated that treatment with erenumab for ≥ 3 months resulted in a ≥ 50% reduction in MMD among 35% of patients, with improvements in migraine severity reported for 45% of patients [31]. In another real-world study evaluating 6-month follow-up data after ≥ 1 erenumab injection, a reduction by 8.4 days in MMD was observed for the 43 patients with available data [16].

This retrospective, panel-based, online physician chart review has several strengths in its design. Its real-world setting allows for an accurate representation of the clinical landscape of migraine. Further, the large sample size of this study allowed for subgroups analyses to be conducted, which sheds light on unique populations with migraine. Additionally, all patients had a physician-confirmed diagnosis of migraine, and healthcare provider and patient-reported outcomes were collected. Together, these yield well-rounded insights into the clinical outlook of migraine.

This real-world study was also subject to certain limitations. While broader insights into the effectiveness of fremanezumab may be gleaned from this study in a real-world setting, the uncontrolled nature of the retrospective study may result in confounding factors that contribute to these differences from the randomized studies [25]. The follow-up period of 6 months was relatively short. In addition, as is typically seen in the real-world setting, patient-reported outcomes, particularly disability scores, were not always reported. Also, as noted previously, follow-up numbers were relatively low, particularly at later timepoints. Reporting rates across different effectiveness outcomes were approximately 19% to 23% by Month 1 in the current study and declined during the course of treatment to approximately 9% to 11% by Month 6. This decrease over time in the proportion of patients who responded to each outcome was likely not due to discontinuation of treatment; over the course of the study, only approximately 8% of participants discontinued fremanezumab treatment. The most likely reason for lower patient response at certain timepoints is that patients are unlikely to have follow-up appointments every month, especially if they are experiencing improvement in their symptoms, with the result that any given monthly timepoint would be expected to capture only a small subset of the total patient population. Other reasons for the low numbers observed at certain timepoints may include physicians’ lack of reporting of the outcomes assessed at all follow-up visits in the charts or survey and the potential that follow-up appointments may have been conducted via telephone, potentially limiting the outcomes captured during those visits. The limited availability of data on these outcomes over the course of treatment suggests a potential area for improvement in patient follow-up [32]; monitoring these outcomes over time helps determine patients’ responses to treatment and can help with assessing any potential need for alterations in patients’ treatment regimens. The number of charts completed by each participating clinician was limited to ≤ 5 to avoid the potential for oversampling by clinicians, which could have introduced bias and reduced generalizability. This did, however, introduce the potential for selection bias because each clinician only selected 5 patient charts meeting inclusion criteria. A randomization scheme was implemented for chart selection to minimize potential bias related to the clinicians’ choice of patient charts for inclusion and to increase generalizability. In addition, this study was conducted in a US population and, due to potential differences in patient characteristics, reimbursement criteria, prescribing practices, and local clinical guidelines, these results may not be generalizable to migraine populations in other countries. Nevertheless, the majority of patients in this population had multiple prior migraine preventive treatment failures (≥ 2 prior failures, 83%; ≥ 3 prior failures, 58%), which is a common criterion for reimbursement in European countries.

The low discontinuation rates and favorable clinical responses observed in this real-world study support the use of fremanezumab as an effective migraine preventive treatment. In other studies with traditional oral migraine preventives [33, 34], patients showed higher rates of discontinuation relative to the current study. In particular, a retrospective cohort analysis comprising patients taking topiramate, β-blockers, or tricyclic antidepressants revealed early gaps in therapy, and 65% of the total cohort had discontinued prophylaxis by the end of the first year [34]. Moreover, a separate retrospective claims analysis in patients with CM found high discontinuation rates by 6 months of therapy that continued to worsen over the course of a year; adherence to therapy ranged between 26% and 29% at 6 months but declined to 17% to 20% at 12 months [33]. In addition to the lower discontinuation rates observed in the current study, patients responded exceptionally well to fremanezumab treatment, as evidenced by the substantial improvements in MMD, MHD, and HIT-6 and MIDAS scores over the course of 6 months. Furthermore, fremanezumab demonstrated comparable efficacy even in patients with more difficult-to-treat migraine, as evidenced in patients with ≥ 2 prior migraine preventive treatment failures, including those with ≥ 4 prior treatment failures.

## Conclusions

The results of this study support the real-world effectiveness of fremanezumab and complement the efficacy previously demonstrated in clinical trials. Additionally, the results described here contribute to the limited data on the real-world use of fremanezumab and other CGRP pathway–targeted monoclonal antibodies [15–19]. The study showed substantial and sustained improvements, after either quarterly or monthly fremanezumab treatment, in MMD, MHD, and HIT-6 and MIDAS scores in a population with migraine, most of whom had previously failed ≥ 2 migraine preventive treatments. Taken together, the results of this real-world study support the effectiveness of fremanezumab as a migraine preventive treatment across a broad population of patients with migraine.

## Supplementary Information

**Additional file 1.** Clinician Characteristics.

**Additional file 2.** Effectiveness outcomes by proportion of patients with ≥30% reduction from baseline in MMD: A) total population; B) dosing schedule subgroups; C) prior treatment failures subgroups.

**Additional file 3.** Effectiveness outcomes by proportion of patients with ≥75% reduction from baseline in MMD: A) total population; B) dosing schedule subgroups; C) prior treatment failures subgroups.

## Data Availability

All data for the analyses presented in this manuscript are included in this published article and its supplementary information files.

## Abbreviations

CGRP: Calcitonin gene-related peptide
CM: Chronic migraine
EM: Episodic migraine
eCRF: Electronic case report form
FDA: US Food and Drug Administration
HIT-6: 6-Item Headache Impact Test
MHD: Monthly headache days
MIDAS: Migraine Disability Assessment
MMD: Monthly migraine days
NHWS: National Health and Wellness Survey
NSAID: Nonsteroidal anti-inflammatory drug
SD: Standard deviation

## References

[1] Feigin VL, Vos T, Alahdab F, Amit AML, Barnighausen TW, Beghi E (2020). Burden of neurological disorders across the US from 1990–2017: a global burden of disease study. JAMA Neurol.

[2] Buse DC, Yugrakh MS, Lee LK, Bell J, Cohen JM, Lipton RB (2020). Burden of illness among people with migraine and ≥ 4 monthly headache days while using acute and/or preventive prescription medications for migraine. J Manag Care Spec Pharm.

[3] Buse DC, Scher AI, Dodick DW, Reed ML, Fanning KM, Manack Adams A (2016). Impact of migraine on the family: perspectives of people with migraine and their spouse/domestic partner in the CaMEO Study. Mayo Clin Proc.

[4] Lipton RB, Lee L, Saikali NP, Bell J, Cohen JM (2020). Effect of headache-free days on disability, productivity, quality of life, and costs among individuals with migraine. J Manag Care Spec Pharm.

[5] American Academy of Neurology. Headache quality management set. 2014. https://www.aan.com/siteassets/home-page/policy-and-guidelines/quality/quality-measures/14headachemeasureset_pg.pdf. Accessed 23 Nov 2021.

[6] Silberstein SD, Holland S, Freitag F, Dodick DW, Argoff C, Ashman E, Quality Standards Subcommittee of the American Academy of Neurology and the American Headache Society (2012). Evidence-based guideline update: pharmacologic treatment for episodic migraine prevention in adults: report of the quality standards subcommittee of the American academy of neurology and the American headache society. Neurology.

[7] Hepp Z, Dodick DW, Varon SF, Chia J, Matthew N, Gillard P (2017). Persistence and switching patterns of oral migraine prophylactic medications among patients with chronic migraine: a retrospective claims analysis. Cephalalgia.

[8] Hepp Z, Bloudek LM, Varon SF (2014). Systematic review of migraine prophylaxis adherence and persistence. J Manag Care Pharm.

[9] Blumenfeld AM, Bloudek LM, Becker WJ, Buse DC, Varon SF, Maglinte GA (2013). Patterns of use and reasons for discontinuation of prophylactic medications for episodic migraine and chronic migraine: results from the Second International Burden of Migraine Study (IBMS-II). Headache.

[10] AJOVY (fremanezumab-vfrm) [prescribing information]. North Wales: Teva Pharmaceuticals USA, Inc. Revised 2021. https://www.ajovyhcp.com/globalassets/ajovy/ajovy-pi.pdf. Accessed 23 Nov 2021.

[11] Dodick DW, Silberstein SD, Bigal ME, Yeung PP, Goadsby PJ, Blankenbiller T (2018). Effect of fremanezumab compared with placebo for prevention of episodic migraine: a randomized clinical trial. JAMA.

[12] Ferrari MD, Diener HC, Ning X, Galic M, Cohen JM, Yang R (2019). Fremanezumab versus placebo for migraine prevention in patients with documented failure to up to four migraine preventive medication classes (FOCUS): a randomised, double-blind, placebo-controlled, phase 3b trial. Lancet.

[13] Silberstein SD, Dodick DW, Bigal ME, Yeung PP, Goadsby PJ, Blankenbiller T (2017). Fremanezumab for the preventive treatment of chronic migraine. N Engl J Med.

[14] Goadsby PJ, Silberstein SD, Yeung PP, Cohen JM, Ning X, Yang R (2020). Long-term safety, tolerability, and efficacy of fremanezumab in migraine: a randomized study. Neurology.

[15] Lambru G, Hill B, Murphy M, Tylova I, Andreou AP (2020). A prospective real-world analysis of erenumab in refractory chronic migraine. J Headache Pain.

[16] Robblee J, Devick KL, Mendez N, Potter J, Slonaker J, Starling AJ (2020). Real-world patient experience with erenumab for the preventive treatment of migraine. Headache.

[17] Kanaan S, Hettie G, Loder E, Burch R (2020). Real-world effectiveness and tolerability of erenumab: a retrospective cohort study. Cephalalgia.

[18] Alex A, Vaughn C, Rayhill M (2020). Safety and tolerability of 3 CGRP monoclonal antibodies in practice: a retrospective cohort study. Headache.

[19] Ornello R, Casalena A, Frattale I, Gabriele A, Affaitati G, Giamberardino MA (2020). Real-life data on the efficacy and safety of erenumab in the Abruzzo region, central Italy. J Headache Pain.

[20] Diener HC, Ashina M, Durand-Zaleski I, Kurth T, Lanteri-Minet M, Lipton RB (2021). Health technology assessment for the acute and preventive treatment of migraine: a position statement of the International Headache Society. Cephalalgia.

[21] Yang M, Rendas-Baum R, Varon SF, Kosinski M (2011). Validation of the Headache Impact Test (HIT-6) across episodic and chronic migraine. Cephalalgia.

[22] Stewart WF, Lipton RB, Dowson AJ, Sawyer J (2001). Development and testing of the Migraine Disability Assessment (MIDAS) Questionnaire to assess headache-related disability. Neurology.

[23] Houts CR, Wirth RJ, McGinley JS, Cady R, Lipton RB (2020). Determining thresholds for meaningful change for the Headache Impact Test (HIT-6) total and item-specific scores in chronic migraine. Headache.

[24] Castien RF, Blankenstein AH, Windt DA, Dekker J (2012). Minimal clinically important change on the Headache Impact Test-6 questionnaire in patients with chronic tension-type headache. Cephalalgia.

[25] Nazha B, Yang JC, Owonikoko TK (2021). Benefits and limitations of real-world evidence: lessons from EGFR mutation-positive non-small-cell lung cancer. Future Oncol.

[26] Pazdera L, Cohen JM, Ning X, Campos VR, Yang R, Pozo-Rosich P (2021). Fremanezumab for the preventive treatment of migraine: subgroup analysis by number of prior preventive treatments with inadequate response. Cephalalgia.

[27] Ashina M, Tepper S, Brandes JL, Reuter U, Boudreau G, Dolezil D (2018). Efficacy and safety of erenumab (AMG334) in chronic migraine patients with prior preventive treatment failure: a subgroup analysis of a randomized, double-blind, placebo-controlled study. Cephalalgia.

[28] Tepper SJ, Ashina M, Reuter U, Brandes JL, Dolezil D, Silberstein SD (2020). Long-term safety and efficacy of erenumab in patients with chronic migraine: results from a 52-week, open-label extension study. Cephalalgia.

[29] Torres-Ferrus M, Gallardo VJ, Alpuente A, Caronna E, Gine-Cipres E, Pozo-Rosich P (2021). The impact of anti-CGRP monoclonal antibodies in resistant migraine patients: a real-world evidence observational study. J Neurol.

[30] Vernieri F, Altamura C, Aurilia C, Brunelli N, Egeo G, Fofi L (2020). Effectiveness, safety, and tolerability of galcanezumab in a real-life setting in patients with migraine in Italy (the GARLIT study). Neurol Sci.

[31] Faust E, Pivneva I, Yang K, Betts KA, Ahmed Z, Joshi S (2021). Real-world treatment profiles, clinical outcomes, and healthcare resource utilization of patients with migraine prescribed erenumab: a multicenter chart-review study of US headache centers. Neurol Ther.

[32] Buse DC, Gillard P, Arctander K, Kuang AW, Lipton RB (2018). Assessing physician-patient dialogues about chronic migraine during routine office visits. Headache.

[33] Hepp Z, Dodick DW, Varon SF, Gillard P, Hansen RN, Devine EB (2015). Adherence to oral migraine-preventive medications among patients with chronic migraine. Cephalalgia.

[34] Woolley JM, Bonafede MM, Maiese BA, Lenz RA (2017). Migraine prophylaxis and acute treatment patterns among commercially insured patients in the United States. Headache.

