# Supplementary material for: Real-world effectiveness of fremanezumab in migraine patients initiating treatment in the United States: results from a retrospective chart study
Source: J Headache Pain. 2022 Apr 11;23(1):47. doi: 10.1186/s10194-022-01411-1 (PMC9004075; doi:10.1186/s10194-022-01411-1)
Supplement: Supplementary file 1 — Additional file 1. Clinician Characteristics. [file 10194_2022_1411_MOESM1_ESM.docx]

**Additional File 1.** Clinician Characteristics

| **Characteristic** | **Clinicians**  **(N = 421)** |
| --- | --- |
| Specialist type, n (%) |  |
| Neurologist | 240 (57.0) |
| General practitioner | 80 (19.0) |
| Pain management specialist | 36 (8.6) |
| Psychiatrist | 21 (5.0) |
| Physician assistant | 21 (5.0) |
| Nurse practitioner | 17 (4.0) |
| Other headache specialist | 6 (1.4) |
| Age, years, n (%) |  |
| 21–30 | 5 (1.2) |
| 31–40 | 146 (34.7) |
| 41–50 | 146 (34.7) |
| 51–60 | 93 (22.1) |
| 61–70 | 30 (7.1) |
| ≥70 | 1 (0.2) |
| Sex, n (%) |  |
| Male | 303 (72.0) |
| Female | 118 (28.0) |
| Time in practice, years, mean (SD) | 14.7 (8.7) |
| US region, n (%) |  |
| South | 131 (31.1) |
| West | 98 (23.3) |
| Midwest | 97 (23.0) |
| Northeast | 95 (22.6) |
| Migraine patients treated in last 12 months, mean (SD) | 367.0 (457.6) |
| Adult patients with migraine treated with fremanezumab, mean (SD) | 68.1 (159.2) |
| Charts per clinician, mean (SD) | 2.4 (1.6) |

SD, standard deviation.
