# Supplementary material for: Real-world effectiveness of fremanezumab in migraine patients initiating treatment in the United States: results from a retrospective chart study
Source: J Headache Pain. 2022 Apr 11;23(1):47. doi: 10.1186/s10194-022-01411-1 (PMC9004075; doi:10.1186/s10194-022-01411-1)
Supplement: Supplementary file 3 — Additional file 3. Effectiveness outcomes by proportion of patients with ≥75% reduction from baseline in MMD: A) total population; B) dosing schedule subgroups; C) prior treatment failures subgroups. [file 10194_2022_1411_MOESM3_ESM.docx]

**Additional File 3.** Effectiveness outcomes by proportion of patients with ≥75% reduction from baseline in MMD**: A)** total population; **B)** dosing schedule subgroups; **C)** prior treatment failures subgroups.


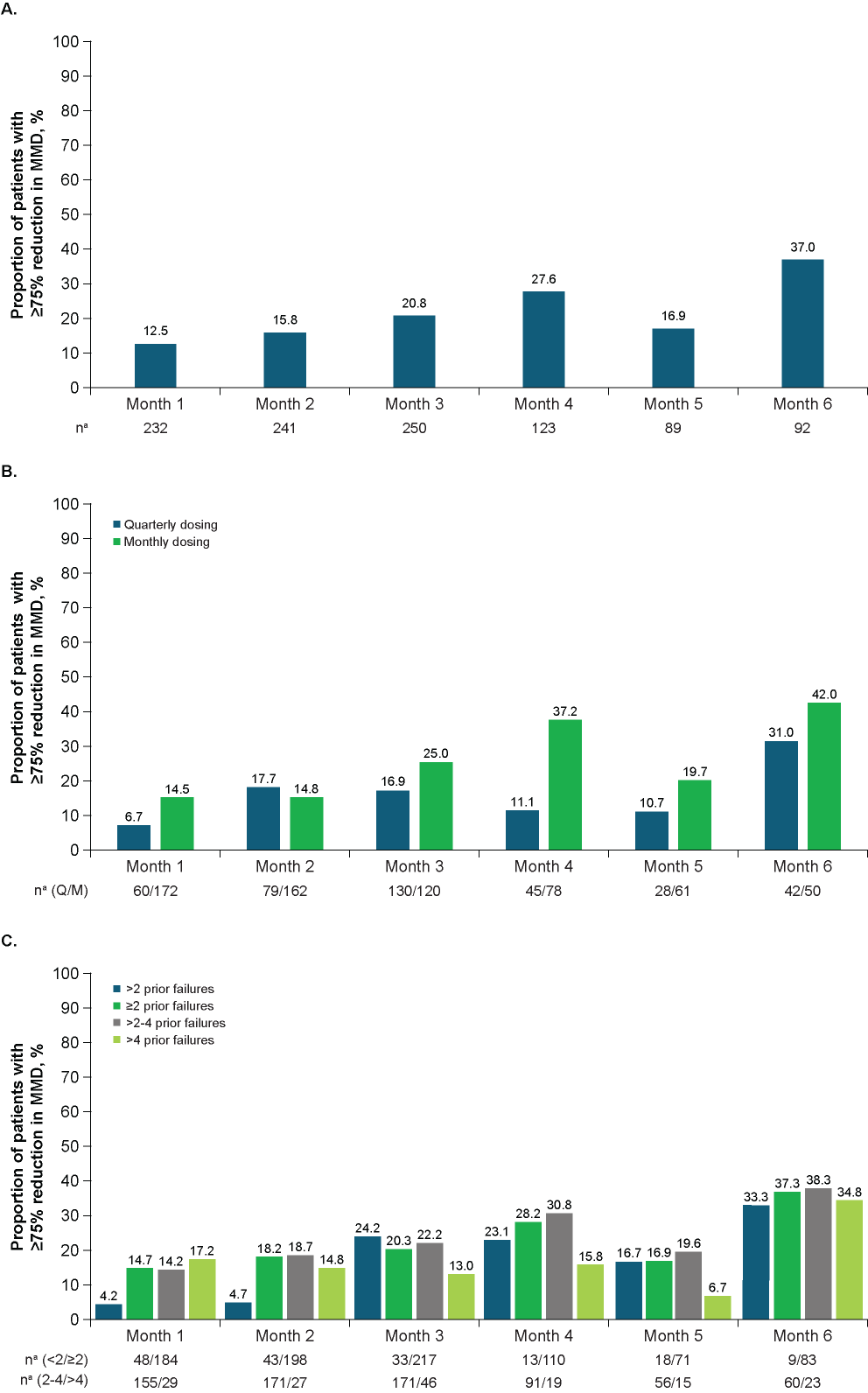


BL, baseline; MMD, monthly migraine days; Q, quarterly; M, monthly.

^a^Number of patients with available assessments at each time point.
